# Sustainable fungi-based protein extraction from agro-waste mushroom stem using deep eutectic solvents

**DOI:** 10.1016/j.fochx.2024.101931

**Published:** 2024-10-24

**Authors:** Gulsah Karabulut, Deniz Günal Köroğlu, Hao Feng, Zekeriya Karabulut

**Affiliations:** a Department of Food Engineering, Faculty of Engineering, Sakarya University, 54050 Sakarya, Türkiye; b Department of Food Engineering, Faculty of Chemical and Metallurgical Engineering, Istanbul Technical University, 34469 Istanbul, Türkiye; c Department of Family and Consumer Sciences, North Carolina A&T State University, Greensboro, North Caroline 27411, USA; d Department of Chemistry, Faculty of Arts and Sciences, Sakarya University, 54050 Sakarya, Türkiye

**Keywords:** Mushroom protein, Deep eutectic solvents, Alternative protein, Functional properties, Green extraction

## Abstract

This study evaluated the nutritional and functional qualities of the mushroom stem (MS) powder and its protein concentrates, which were extracted using alkaline and deep eutectic solvent (DES)-based methods. MS powder is rich in protein (23.02 %) and carbohydrates (63.01 %). Alkaline extraction yielded the highest protein content (45.90 %) but lower recovery rates (15.39 %) compared to DES extractions using a 1:2 molar ratio of choline chloride: glycerol (DES-CG) and choline chloride: lactic acid (DES-CL), which had recovery rates of 22.04 % and 21.47 %, respectively. DES solvents provided strong hydrogen bonding, as indicated by FTIR analysis, and produced uniform, smooth particles according to SEM imaging. Both DES and alkaline methods achieved similar protein solubility values (72–75 %). However, DES methods better preserved emulsion capabilities and balanced foaming properties, enhancing foam capacity and maintaining stability. The findings suggest that DES methods are more sustainable and efficient for extracting proteins from MS powder, optimizing both functional properties and environmental sustainability in food applications.

## Highlights

• DES extraction shows strong hydrogen bonding and effective protein retrieval.
• Alkaline extraction yields highest protein content but lowest recovery rate.
• DES methods improve protein solubility, enhancing lignocellulosic disruption.
• DES extraction balances yield and functionality, adhering to green principles.

## Graphical abstract

**Figure:**
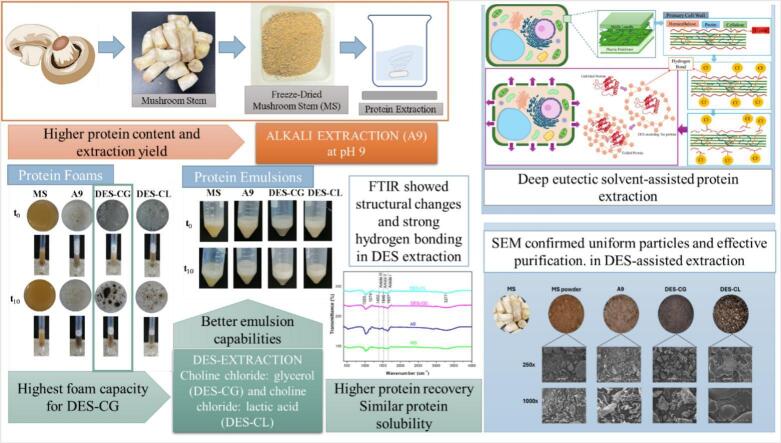

## 1 Introduction

Globally, an estimated one-third of the food produced for human consumption, approximately 1.3 billion tons annually, is lost or wasted. Among these reasons, the prevalent attitude in industrialized nations is that throwing away is cheaper than using or reusing (FAO, 2011). In response to this challenge, the FAO (2023) launched the “*Towards a Waste-Free Future*” strategy, which focuses on enhancing the efficient use of existing biological resources and minimizing food waste through improved resource management and sustainability practices.

Recently, plant and fungal proteins, particularly those sourced from food waste, have gained significant attention as sustainable alternatives to animal proteins. These proteins are extensively researched for their functional properties, anti-allergic potential, and cost-effectiveness in producing meat analogs and present significant advantages (Wang & Zhao, 2023). Effective utilization of these proteins involves selecting the most suitable (optimal) extraction methods that maximize protein yield while preserving their functional qualities.

While thousands of mushroom species exist in the wild, only 25 are considered edible (Antunes et al., 2020). *Agaricus bisporus* is the most commonly consumed, comprising 30 % of the global edible mushroom market (Bermúdez-Gómez et al., 2024). Edible mushrooms are recognized for their nutritional value, rich in proteins, minerals, and dietary fibers yet low in calories and fats. They are considered a high-quality protein source, characterized by a complete essential amino acid score (EAAS>1) and branched-chain amino acids similar to those found in animal proteins. Additionally, they exhibit high protein digestibility (60–70 %) and protein digestibility corrected amino acid score (PDCAAS: 0.35–0.70) (Ayimbila & Keawsompong, 2023; Wang & Zhao, 2023).

Edible mushrooms are increasingly utilized in diets as a source of high-quality plant-based protein. Their production and consumption are driven by nutritional benefits and global popularity. However, the mushroom production process generates significant by-products such as caps, strips, corks, and spent mushroom substrate, constituting approximately 20 % of the total production volume (Antunes et al., 2020; Umaña et al., 2020). Notably, MS, a major by-product, is nutritionally valuable. It is rich in dietary fiber (primarily insoluble), ergosterol (a provitamin), and phenolic compounds, and contains proteins that are abundant in amino acids contributing to an umami flavor, predominantly 5’-GMP (Guanosine monophosphate) (Banerjee et al., 2020; Bermúdez-Gómez et al., 2024; Garcia et al., 2023; Prandi et al., 2023; Umaña et al., 2020). Nonetheless, MS is tough to chew, swallow, and digest, making it commonly discarded during harvesting and food processing. However, they can be used in two main ways: dried, crushed, and added to food or feed to reduce costs and increase fiber content, or processed to extract valuable compounds like protein, polysaccharides, polyphenols, and flavonoids (Guo et al., 2022).

There are several reseach on the extraction of nutrients from *Agaricus bisporus* stems (Al-Mahmood & al Mudhaffar, 2022; Rajabzadeh Shandiz et al., 2019; Shahadha et al., 2023; Wu et al., 2004). Recent studies have focused on mannose-binding proteins (Ismaya et al., 2021; Nabila et al., 2019; Rachmawati et al., 2019) and the interaction of carbohydrates with lectin (He, Condict, Richardson, Brennan and Kasapis, 2024, He, Condict, Richardson, Brennan and Kasapis, 2025) in *A. bisporus*. Wang et al. (2024) identified peptides from enzymatically hydrolyzed *A. bisporus* protein that enhance saltiness. Among other studies, the bioactive properties of *A. bisporus* proteins hydrolyzed with alcalase or pepsin enzymes have been investigated (Farzaneh et al., 2018; Izanloo et al., 2022; Izanloo & Sadeghi Mahoonak, 2023). Although mushroom proteins are utilized as non-animal protein sources in foods, there is currently a limited number of commercial productions of protein concentrates or isolates from the waste or whole mushrooms. Ragucci et al. (2023) characterized a protein from *A. bisporus*, bisporitin, which exhibits magnesium-dependent endonuclease activity. Bisporitin demonstrated low thermal stability (Tm = 48.59 ± 0.98 °C) relative to other ribotoxin-like proteins (above 70 °C). In simulated digestion experiments, bisporitin was only partially degraded without adversely affecting eukaryotic cell lines.

Alkaline extraction, widely employed in the food industry, facilitates high extraction yields by solubilizing proteins and removing insoluble non-protein materials under highly alkaline conditions. However, choosing an inappropriate pH during alkaline extraction can lead to protein denaturation, the formation of undesirable complexes, or induce toxic effects (Hadidi et al., 2023). Alkaline-based protein extraction is a common and straightforward method, but it can negatively impact protein quality by altering protein structures, causing polyphenol oxidation, and forming lysinoalanine, which reduces protein digestibility and loses essential amino acids. Furthermore, deep eutectic solvents (DES) are composed of ionic or non-ionic compounds in specific ratios, requiring heating, stirring, or sonication to reach a eutectic point. They are eco-friendly, biodegradable, non-toxic, non-flammable, and sustainable, with low vapor pressure and reduced volatility. In contrast, traditional extraction solvents such as ionic liquids, organic solvents, and alkaline solutions like sodium hydroxide can be toxic, flammable, volatile, and pose environmental concerns due to poor biodegradability and hazardous waste generation (Patra et al., 2023). Additionally, the process generates residual wastewater during the acid-neutralization stage. Despite its widespread use, there is limited research on the environmental sustainability of alkaline-mediated protein extraction. DES offers several advantages, including ease of preparation, minimal purification, and their ability to disrupt cell walls and enhance protein solubility, making them highly efficient for bioactive compounds and protein extraction. Their low volatility, high solvation power, and environmentally friendly nature make them a promising alternative to conventional solvents, supporting the transition toward more efficient and sustainable processing with a lower environmental footprint (Hanafi et al., 2024).

DESs effectively extracted proteins from sources like sesame meal, pomegranate peel, brewer's spent grain, and *Sacha inchi* meal biomass (Hernández-Corroto, Plaza, Marina and García, 2020, Hernández-Corroto, Olivares-Galván, Marina and García, 2023; Sharma et al., 2023; Wahlström et al., 2017), with ultrasound-assisted DES extraction, particularly using choline chloride (ChCl): glycerol, enhancing protein solubility and digestibility (Sharma et al., 2023). They also selectively extracted proteins from brewing residues, avoiding phenolic compounds and improving the dissolution of insoluble or denatured proteins (Hernández-Corroto et al., 2023; Wahlström et al., 2017). As an emerging technology, DES-based protein extraction is still in its early stages, requiring further research to understand its mechanisms and impact on the functional properties of the extracted proteins.

This research aimed to extract protein from MS powder as a sustainable alternative protein source. For extraction, two methodologies were employed: traditional alkaline extraction and eco-friendly DES-assisted extractions (ChCl: glycerol or ChCl: lactic acid). The extraction yield (%) and protein recovery rate (%) of MS proteins were compared. Furthermore, a comparative analysis of the functional properties (such as solubility, foaming capacity, and emulsification properties), physicochemical properties, and morphological characteristics of the protein extracts obtained from these two extraction methods was conducted. This evaluation enabled the identification of the most effective extraction method for preserving the desirable properties of proteins derived from MS while presenting greener extraction alternatives.

## 2 Materials and methods

In this study, locally sourced MS from *A. bisporus*, typically considered waste by a factory specializing in the production of canned sliced mushrooms, was utilized as a raw material. This approach aimed to explore the potential for valorizing these by-products, thereby contributing to waste reduction and promoting more sustainable food production practices. Impurities were removed by hand from MS, and then they were freeze-dried (Labconco, USA). Dried MS was ground by a blender (Waring 8011 EB, USA) and passed through 40 mesh sieves to obtain a uniform MS.

The composition of the MS powder in this study was analyzed using the procedures established by the Association of Official Analytical Chemists (AOAC International, 1990). Protein contents of MS powder and extracted proteins were determined using a Kjeldahl Nitrogen analyzer (Behr Labor Technik, Germany) with a nitrogen conversion factor of 6.25. Carbohydrate content was calculated using the following formula (Eq. 1):

(1) $$\mathrm{Carbohydrate}\;\left(\%\right)=100-\left(\mathrm{Protein}+\mathrm{Lipid}+\mathrm{Ash}+\mathrm{Moisture}\right)$$

### 2.1 Protein extraction from mushroom stem powder

#### 2.1.1 DES-assisted protein extraction

**Fig. 1:**
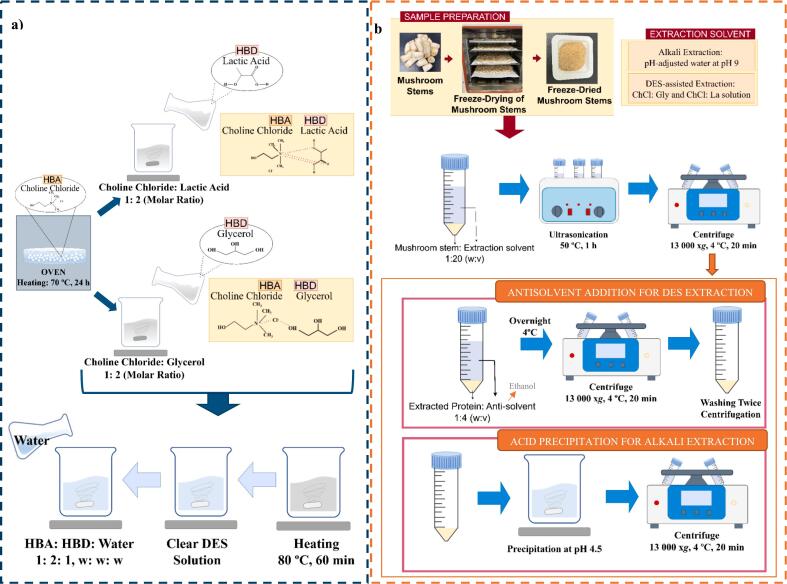
(a) Deep eutectic solvent (DES) formation steps. HBA: Hydrogen bond acceptor; HBD: hydrogen bond donor (b) DES or alkaline extraction of mushroom stem protein.

DES formation steps are shown in Fig. 1a. ChCl was dried at 70 °C for 24 h before use. Two types of DES were prepared by mixing ChCl (hydrogen bond acceptor, HBA) with either glycerol (Gly) or lactic acid (La) (hydrogen bond donor, HBD) in a 1: 2 molar ratio at ambient temperature for 30 min, followed by heating at 80 °C for 60 min until a clear and colorless solution was formed. The DES solutions remained in a clear liquid state after 24 h at room temperature. The resulting solutions were then diluted with distilled water to achieve a 75 % DES/25 % water concentration, reducing the viscosity of the DES (Sharma et al., 2023).

MS powder in DES solutions at a 1: 20 (w: v) ratio was extracted using an ultrasonic (US) bath (35 kHz, 640 W) (Bandelin, Sonorex Super, Germany) at 50 °C for 1 h, followed by centrifugation (Hettich, Universal 320R, Germany) at 13,000 x*g* and 4 °C for 20 min. The supernatant was collected. Protein recovery was achieved via antisolvent (ethanol) addition. For antisolvent precipitation, ethanol was added to the protein extract at 4 times its volume, and the mixture was stood at 4 °C overnight. The mixture was then centrifuged at 13,000 x*g* and 4 °C for 20 min, and the resulting pellet was collected. The pellet was washed twice with distilled water, centrifuged, and then freeze-dried (Fig. 1b). Proteins extracted using DES, i.e., ChCl: Gly and ChCl: La were labeled as DES-CG and DES-CL, respectively.

#### 2.1.2 Alkaline extraction of mushroom stem protein

MS powder (1: 20, w: v) was dissolved in water adjusted to pH 9. The extraction at pH 8, 9, and 10 was preliminarily tested based on the protein content in the extracts, with pH 9 yielding the highest protein content (Data not shown). The suspension was extracted in a US bath (35 kHz, 640 W) (Bandelin, Sonorex Super, Germany) at 50 °C for 1 h and then centrifuged at 13,000 x*g* at 4 °C for 20 min. The obtained supernatant was collected and precipitated at pI 4.5 (isoelectric point) after centrifugation (Fig. 1b). The pellet was freeze-dried. Proteins extracted under alkaline conditions at pH 9 were labeled as A9.

### 2.2 Protein content of aqueous protein extracts

Total soluble protein content was analyzed using the Bradford method (Bradford, 1976). A 150 μL aliquot of protein solution (10 mg/mL in distilled water) was mixed with 3 mL of Bradford reagent solution. After 10 min, absorbance values were recorded at 595 nm using a UV–Vis spectrophotometer (Shimadzu, UVMINI 1240, Japan) against a blank, which consisted of 150 μL of distilled water instead of the protein solution. Protein content was calculated from a calibration curve generated with bovine serum albumin (BSA) standards (10 to 200 μg/mL, *R*^*2*^ 0.995).

### 2.3 Extraction yield (%) and protein recovery rate (%) determination

Extraction yield and protein recovery were calculated to identify the efficiency of protein extraction methods from the MS powder. By determining the protein content in proteins and MS powders, the extraction yield and protein recovery rate were calculated as a percentage (%) in Eq. (2), (3).

(2) $$\mathrm{Extraction yield}\;\left(\%\right)=\frac{M_{\mathrm{MSP}}}{M_{\mathrm{MS}}}\times 100\;$$

(3) $$\mathrm{Protein recovery rate}\;\left(\%\right)=\frac{M_{\mathrm{MSP}}{\mathrm{xP}}_{\mathrm{MSP}}}{M_{\mathrm{MS}}{\mathrm{xP}}_{\mathrm{MS}}}\times 100\;$$

where *P*_*MSP*_ is the protein content, $M_{\mathrm{MSP}}$ is the weight of MS protein, *P*_*MS*_ is the protein content and $M_{\mathrm{MS}}$ is the weight of MS powder.

### 2.4 Fourier transform infrared spectrophotometer (FTIR)

FTIR spectra of protein powders (∼10 mg) were acquired via attenuated total reflection, at the range of 400–4000 cm^−1^, using an FTIR spectrometer (Perkin Elmer Spectrum Two, USA) under ambient conditions. Each spectrum was obtained with 32 scans at a resolution of 4 cm^−1^. The background spectrum of air was subtracted from the sample spectrum using the software FTIR spectrometer (Perkin Elmer Spectrum Two, USA).

### 2.5 Morphological properties

Scanning electron microscopy (Jeol, JSM 6060 LV, Japan) was used to assess alterations in the morphology of protein samples. The samples were coated with gold/palladium and inspected at voltages of 15 kV, with magnifications of 250× and 1000 × .

### 2.6 Solubility

The solubility of protein was determined by modifying the method of Morr et al. (1985). Briefly, MS powder and proteins were dissolved in water (10 mg/mL, pH 7) and stirred at 450 rpm for 1 h at ambient conditions. After centrifugation (13,000 x*g*, 20 min, 25 °C), the protein content in the supernatant was determined by the Bradford method. Protein solubility (%) was calculated according to the following Eq. 4.

(4) $$\mathrm{Protein solubility}\left(\%\right)=\frac{P_{\mathrm{soluble}}}{P_{\mathrm{total}}}\;$$

where *P*_*soluble*_ and *P*_*total*_ are protein content in supernatant and analyzed protein content from Kjehldahl method.

### 2.7 Emulsion properties

Emulsion activity (EA) and stability (ES) indices of MS powder and protein extracts were measured by modifying the method used by Pearce and Kinsella (1978). A 4 mL of protein suspension in distilled water (5 mg/mL, pH 7.0) was homogenized with 1 mL of sunflower oil using a homogenizer (IKA, T18, Königswinter, Germany) for 1 min at 20,000 rpm. After homogenization, 25 μL of the emulsion was taken from the bottom of this homogenate and then diluted with 2.5 mL of sodium dodecyl sulfate solution (SDS) (0.1 % *w*/*v*). EA was determined by measuring the absorbance at 500 nm of the diluted emulsion using a UV–Vis spectrophotometer (Shimadzu, UV-1240, Kyoto, Japan). ES was determined by measuring the emulsion absorbance after 10 min. EA and ES were calculated according to Eq. (5) and (6).

(5) $$\mathrm{EA}\;\left(m^{2}/g\right)=\frac{2*2.303*A_{0}*\mathrm{DF}}{c*\emptyset *\partial *10000}\;$$

(6) $$\mathrm{ES}\;\left(\min\right)=\frac{A_{0}*\mathrm{\Delta t}}{A_{0}-A_{10}}\;$$

where *DF* is the dilution factor, c is the initial concentration of protein (g/mL), ∅ is the optical path (1 cm), Δt is equal to 10 min, and ∂ is the fraction of oil used to form the emulsion (mL/mL), whereas *A*_0_ and *A*_10_ are the absorbances of the diluted emulsions after 0 min and 10 min, respectively.

### 2.8 Foaming properties

Foaming capacity (FC) and stability (FS) were determined according to the method described by Karabulut and Feng (2024) with minor modifications. For foam formation, protein suspensions (10 mL) in distilled water (5 mg/mL, pH 7.0) were homogenized at 20,000 rpm for 1 min using a homogenizer. FC and FS were calculated using Eq. (7) and (8).

(7) $$\mathrm{FC}\;\left(\%\right)=\frac{F_{0}}{V_{0}}x100\;$$

(8) $$\;\mathrm{FS}\;\left(\%\right)=\frac{F_{10}}{F_{0}}x100\;$$

where $F_{0}\;$and $F_{10}\;$are the foam volume at 0 min and 10 min. $V_{0}$ is the volume of initial protein suspensions.

### 2.9 Statistical analysis

Analysis results were evaluated using the SPSS 20.0 package program (SPSS Inc., Chicago, USA). Analyzes were performed in triplicate and the results were given as mean ± standard deviation. The difference between mean values was determined within a 5 % confidence interval using ANOVA and Tukey's multiple comparison test.

## 3 Results and discussions

The proximate composition of MS powder provides valuable insights into its nutritional profile, revealing the following components: moisture (3.67 %), crude protein (23.02 %), ash (8.15 %), fat (2.15 %), and carbohydrates (63.01 %) (Table 1). The significant protein content of MS underscored its potential as a substantial protein source. Furthermore, the moderate ash content indicated a beneficial presence of minerals, while the low-fat percentage makes it an excellent option for low-fat dietary preferences. These findings were consistent with those reported by Banerjee et al. (2020), who identified the proximate composition of MS as having a protein range of 12.75–18.42 %, fat content between 1.5 and 2.94 %, and ash content ranging from 6.33 to 11.6 %. The observed variations between studies can be attributed to differences in harvesting methods, stages of maturity, soil types, and environmental conditions. Additionally, Baziuon et al. (2020) reported the protein content of white button MS at 16.8 %, with variations in other studies also attributed to differences in the nitrogen conversion factor, which ranges between 4.38 and 6.25. Overall, the balanced composition of macronutrients and minerals in MS powder underscores its suitability for a variety of dietary requirements, making it a versatile and nutritious ingredient.

**Table 1:** Proximate composition of the mushroom stem powder.

Component (%)	Mushroom Stem
Moisture	3.67 ± 0.23
Crude protein	23.02 ± 1.61
Ash	8.15 ± 0.94
Fat	2.15 ± 0.12
Carbohydrate	63.01 ± 2.15

### 3.1 Extraction yield (%) and protein recovery rate (%)

The protein content, extraction yield, and protein recovery rate from MS are shown in Table 2. The extract obtained by alkaline extraction (A9) demonstrated the highest protein content at 45.90 %, comparable to DES-CL at 42.51 % and significantly higher than DES-CG at 34.45 %. Additionally, A9 showed the highest extraction yield at 12.21 %, outperforming DES-CG (5.69 %) and DES-CL (5.71 %). However, DES-CG and DES-CL exhibited higher protein recovery rates of 22.04 % and 21.47 %, respectively, compared to 15.39 % of A9, suggesting that DES-CG and DES-CL were more effective in preserving protein integrity relative to the initial content. The results could also be due to the better selectivity of DES for protein extraction (Lin et al., 2021; Ozel & Elibol, 2024). DES-CL exhibits greater polarity due to the presence of carboxylic acid groups in lactic acid and has lower viscosity compared to DES-CG.

**Table 2:** Extracted protein content (%), extraction efficiency (%), and protein recovery (%) from mushroom stems using alkaline and DES-based methods.

Samples⁎	Protein content (%)	Extraction yield (%)	Protein recovery rate (%)
A9	45.90 ± 0.81^a^	12.21 ± 2.18^a^	15.39 ± 0.95^b^
DES-CG	34.45 ± 4.04^b^	5.69 ± 0.66^b^	22.04 ± 1.90^a^
DES-CL	42.51 ± 2.49^a^	5.71 ± 0.65^b^	21.47 ± 1.05^a^

⁎ Alkaline-based extract/protein at pH 9.0: A9, DES-based extracts/proteins ChCl: glycerol: DES-CG and ChCl: lactic acid: DES-CL. The column values with different alphabets are significantly different (*p* < 0.05).

**Fig. 2:**
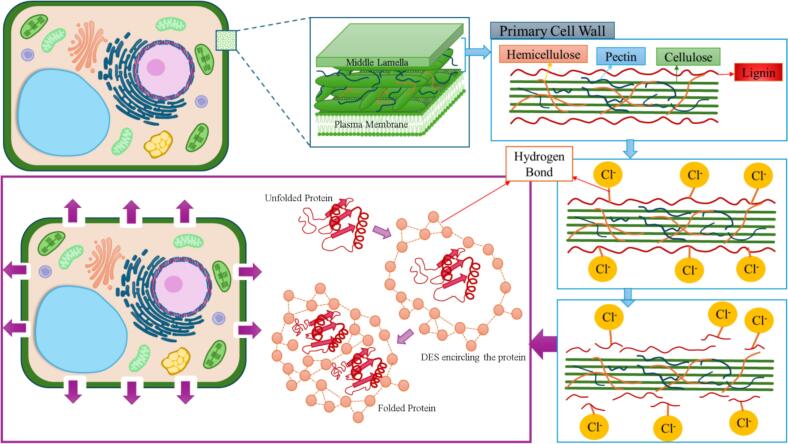
Supposed mechanism of deep eutectic solvent-assisted protein extraction from lignocellulosic biomass.

The extraction of proteins from MS involves overcoming the robust lignocellulosic structure, mainly composed of cellulose, hemicellulose, and lignin (Fig. 2). In particular, chloride anions (Cl^−^) from ChCl form hydrogen bonds with lignin, aiding in the breakdown of ester or ether bonds between hemicellulose and lignin, promoting lignin degradation (Ong et al., 2021). DESs, made from an HBD and an HBA, form a eutectic mixture with a lower melting point than their individual components, aiding in the disruption of the lignocellulosic structure and enhancing protein accessibility (Zhou et al., 2022). For example, Lin et al. (2021) found that DES extraction of bamboo shoot protein resulted in higher protein recovery and effectively prevented amino acid denaturation and alteration at elevated pH levels. Similarly, Hewage et al. (2024) reported that using a ChCl:Gly for fava bean protein extraction achieved a protein content of 92.33 %, a yield of 65.42 %, and a protein recovery rate of 23.15 %, outperforming traditional alkaline extraction methods in terms of yield (60.76 %) and recovery rates (21.74 %). Yue et al. (2021) achieved a protein content of 55.82 %, an extraction yield of 11.75 %, and a recovery rate of 41.43 % using ChCl-dihydric alcohol-based DES for oat protein extraction.

The mechanism of protein extraction using DESs involves multiple interactions: hydrogen bonding between the HBD component and protein amino acid residues, electrostatic interactions between the ionic DES components and charged amino acid residues, and hydrophobic interactions that assist in protein solubilization (Hanafi et al., 2024). This multi-faceted approach enables DESs to extract proteins selectively, minimizing the co-extraction of non-target compounds and preserving the native structure of the proteins. Studies suggest that DESs, composed of osmolyte-like components, may stabilize protein structures in solution, similar to osmolytes that protect cells from osmotic shock. This effect suggests that DESs might induce denatured proteins to refold into soluble configurations through a “crowding” effect (Hanafi et al., 2024; Wahlström et al., 2017; Yue et al., 2021). In contrast, alkaline extraction utilizes high pH solutions, typically sodium hydroxide, to hydrolyze ester and amide bonds in cell wall components, leading to cell wall breakdown and protein release. However, the high pH environment often causes protein denaturation by disrupting hydrogen bonds, hydrophobic interactions, and disulfide bonds, which can be detrimental when native protein functionality is desired (Kim et al., 2021).

### 3.2 FTIR

**Fig. 3:**
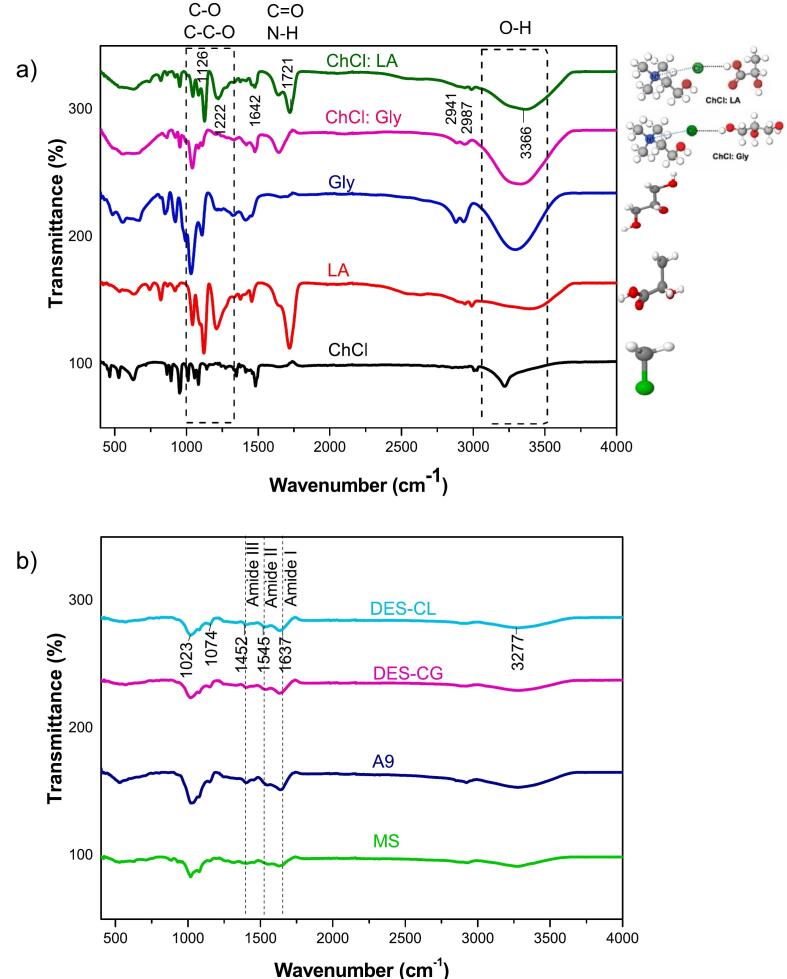
FTIR graph of a) deep eutectic solvent components and mixtures and b) mushroom stem powder and alkaline and DES-based extracted proteins. ChCl: choline cloride, ChCl: Gly, choline cloride: glycerol, ChCl: LA: choline chloride: lactic acid, MS: mushroom stem, A9: alkaline-extracted protein, DES-CG: ChCl: glycerol-based extracted proteins, and DES-CL: choline chloride: lactic acid-based extracted proteins.

FTIR spectra in Fig. 3 demonstrate the characteristics of ChCl as an HBA and its combinations with Gly and La as HBD to form DES. The spectrum of ChCl illustrates characteristic peaks, including a possible C—N stretch from its quaternary ammonium group, with additional features potentially due to the chloride ion. La exhibits a broad O—H stretch from 2500 to 3500 cm^−1^ and a distinct C=O stretch near 1700 cm^−1^, with C—O stretching vibrations evident around 1000 to 1300 cm^−1^. The Gly spectrum similarly displays broad O—H stretch and specific C—O stretch vibrations, typical for polyols. The DES spectra for ChCl: La and ChCl: Gly show distinct modifications in peak patterns when compared to the individual components. These changes include reduced O—H stretch intensity and shifted C=O stretch, indicative of strong hydrogen bonding and intermolecular interactions, confirming DES formation.

The FTIR spectrum provides insights into the structural composition of MS and its protein powders obtained through different extraction methods, including alkaline extraction (A9) and DES-assisted extraction (DES-CG and DES-CL). The MS spectrum shows characteristic peaks at 3300–3400 cm^−1^, indicating O—H stretching typical of polysaccharides and proteins, a peak around 2900 cm^−1^ corresponding to C—H stretching, and prominent amide I (1640 cm^−1^) and amide II (1540 cm^−1^) bands, indicative of protein content. Additionally, peaks in the 1400–1300 cm^−1^ range suggest the presence of alkanes or proteins. The A9 spectrum reveals more defined peaks at 1640 cm^−1^ and 1540 cm^−1^, suggesting increased protein content and possibly different secondary structures compared to DES-CG and DES-CL. Slight shifts in the 2900 cm^−1^ and 1400–1300 cm^−1^ regions indicate alterations in lipid or polysaccharide content. The DES-CG spectrum shows a strong, broad peak around 3300–3400 cm^−1^, reflecting enhanced extraction of proteins with significant hydrogen bonding. Prominent amide I and II peaks confirm substantial protein extraction, while variations in the 1400–1300 cm^−1^ region imply changes in protein structures. Similarly, the DES-CL spectrum exhibits a broad O—H stretching peak and strong amide I and II bands, indicating high protein extraction efficiency. Variations in the fingerprint region (1500–500 cm^−1^) suggest the presence of unique compounds extracted using DES. A similar trend was reported by Hewage et al. (2024) for DES-extracted fava bean proteins. Additionally, Chen et al. (2021) investigated the FTIR spectral differences between commercial and DES-extracted soybean proteins. Comparatively, alkaline extraction shows clear protein peaks, indicating effective protein extraction but possibly different from DES methods in terms of protein structure and secondary components. Both DES-CG and DES-CL are highly effective in extracting proteins and additional hydrogen-bonded components, suggesting a more comprehensive extraction process.

### 3.3 Morphological properties

**Fig. 4:**
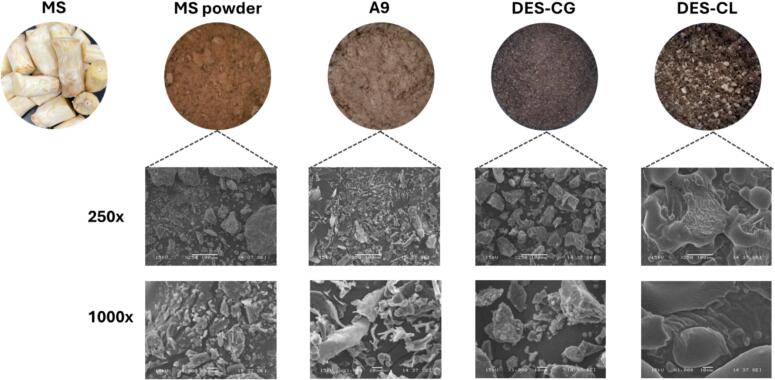
SEM images of mushroom stem proteins. MS: mushroom stem, A9: alkaline-extracted protein, DES-CG: ChCl: glycerol-based extracted proteins, and DES-CL: choline chloride: lactic acid-based extracted proteins.

SEM images in Fig. 4 provide insight into the morphological changes of MS-derived protein powders via various extraction methods.

The MS powder exhibited irregularly shaped particles with a rough and fibrous surface texture, indicating its heterogeneous nature. In contrast, the A9 proteins displayed more uniform particles with smoother surfaces, suggesting the partial removal of fibrous components during extraction. A9 particles showed defined structures with reduced porosity, indicative of a higher protein concentration and a decrease in non-protein materials. The DES-extracted protein powders, specifically those extracted using DES-CG and DES-CL, demonstrated further refinement. Both DES-CG and DES-CL samples exhibited fairly uniform particles with smooth surfaces, highlighting the efficiency of these solvents in protein extraction and purification. However, the DES-CG sample presented a compact and smooth surface, suggesting dense protein aggregates, whereas the DES-CL sample revealed a slightly more porous surface texture, likely due to the specific interactions between lactic acid and proteins during extraction. Our observation agrees with Hewage et al. (2024) findings. SEM analysis illustrates that both alkaline and DES extractions significantly enhance the uniformity and smoothness of the MS-derived protein powders, with DES methods showing a slight variation in surface porosity depending on the solvent composition used.

### 3.4 Solubility

**Fig. 5:**
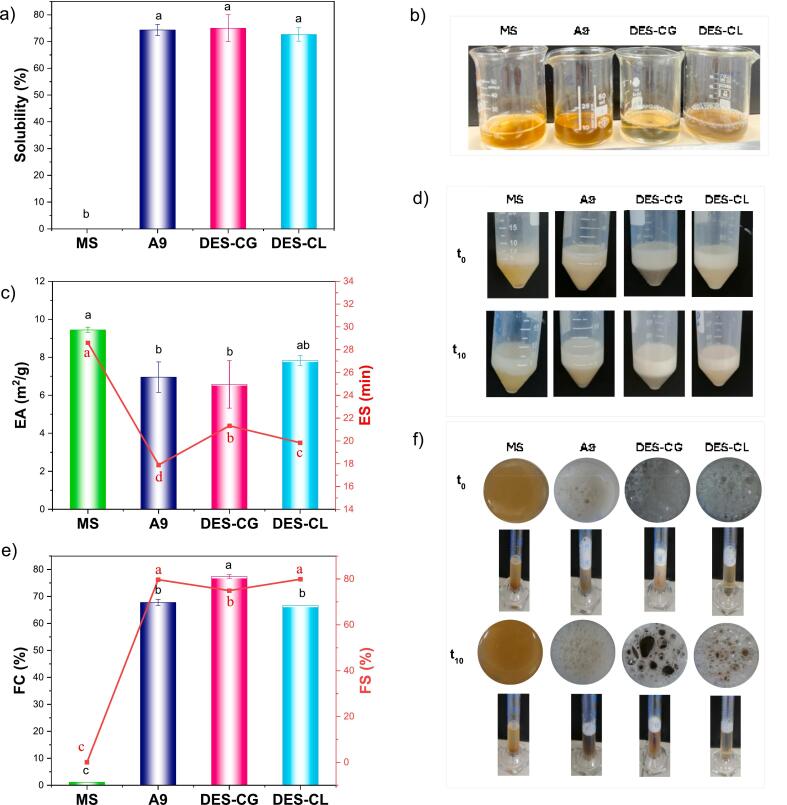
a) Solubility, b) soluble suspension photos, c) emulsion activity and stability, d) emulsion photos, e) foaming capacity and stability, and f) foam photos of the mushroom stem proteins. MS: mushroom stem, A9: alkaline-extracted protein, DES-CG: ChCl: glycerol-based extracted proteins, and DES-CL: Choline chloride: lactic acid-based extracted proteins.

Solubility is an indicative of protein denaturation and aggregation, and fundamentally influences all other functional properties. High solubility is essential for optimal functionality, as it exposes active protein groups. The solubility of proteins extracted from MS using various methods is depicted in Fig. 5a-5b. Both alkaline and DES-based extraction methods showed similar solubility values, ranging between 72 and 75 %.

The DES-CG and DES-CL methods underscore the efficacy of DES in disrupting biomass, particularly within the rigid structure of MS. The elevated solubility metrics observed with DES methods indicate a profound disruption of the cellular matrix, facilitated by the solvent's capacity to permeate and degrade lignocellulosic barriers, significantly enhancing protein release and solubility. DES solvents, by forming hydrogen bonds with proteins, create a network that surrounds the proteins (Fig. 2) without causing chemical changes, allowing the recovery of proteins in their natural, undistorted state (Patra et al., 2023). According to Yue et al. (2021), DES can modify amino acid profiles and hydrophilic/hydrophobic segments due to their selectivity for specific subunits of oat protein, resulting in a higher proportion of hydrophilic residues (approximately 58 %). Moreover, DES methods are effective and sustainable options for extracting proteins from biomass.

### 3.5 Emulsion properties

Fig. 5c-5d illustrates the EA and ES among MS and different extracted protein samples. The data reveal that MS powder outperforms proteins extracted using other methods, exhibiting the highest EA (9.44 m^2^/g) and ES (28.61 min). This indicates that the polysaccharides and proteins in mushrooms enhance the viscosity of the aqueous phase, further stabilizing the emulsion. The A9 extraction method shows the most significant decrease in both EA (6.95 m^2^/g) and ES (17.89 min), likely due to the harsh alkaline conditions causing denaturation or alteration of protein structures, adversely affecting their emulsifying functionalities. Similarly, proteins extracted with DES methods, though slightly better in retaining emulsion capabilities (ES values of 21.32 min for DES-CG and 19.83 min for DES-CL), still fall short of the performance exhibited by the MS powder.

These findings suggest that while DES extraction is less damaging than alkaline extraction, it also leads to some structural modifications that diminish protein functionality in emulsion systems. The emulsifying ability of MS proteins is significantly influenced by the extraction method. Alkaline extraction uses high pH to denature proteins by disrupting hydrogen bonds and disulfide bridges, leading to protein unfolding and aggregation (Karabulut, Goksen and Khaneghah, 2024a, Karabulut, Kapoor, & Feng, 2024b). This structural change often reduces the effectiveness of proteins in stabilizing emulsions due to inconsistent exposure to hydrophobic and hydrophilic sites. In contrast, DES methods interact with proteins less destructively, preserving more native secondary and tertiary structures, which enhances emulsifying properties (Hewage et al., 2024). DES-specific interactions can be tailored to optimize these properties, whereas alkaline solutions can cause extensive chemical modifications like deamidation, further diminishing protein functionality in emulsion systems.

### 3.6 Foaming properties

The foaming properties of MS powder and its proteins extracted via alkaline and DES-based methods show significant differences, as illustrated in Fig. 5e-5f. MS powder has a low FC of 1.11 %, indicating that its lignocellulosic complex form does not effectively trap air, but it is effective at stabilizing it. The A9 method significantly enhances FC to 67.78 %, likely by unfolding proteins and exposing hydrophobic groups necessary for stabilizing air bubbles. However, this reduces FS to 79.69 %, possibly due to over-unfolding impairing the structural integrity of the foam.

Alkaline extraction enhances FC by unfolding proteins but reduces FS and ES, implying that the process improves initial foam and emulsion formation but compromises long-term stability due to structural weakening. DES-CG yields a higher FC of 77.41 % compared to alkaline extraction (67.77 %), suggesting this DES solvent may preserve or enhance structures conducive to foaming. FS values for DES-CL and A9 (79.89 % and 79.69 %) are higher than DES-CG (74.95 %), indicating a better balance between foam capacity and stability. The greater foaming capacity of DES-CG may be attributed to the higher polarity and lower viscosity of DES-CL, which is due to the presence of carboxylic acid groups in DES-CL.

This suggests that while alkaline extraction effectively increases foam capacity, it compromises stability, whereas DES methods offer a balanced approach by moderately altering protein structures, supporting both foam formation and stability (Karabulut et al., 2024c). The higher protein content in A9 contributes to its enhanced foaming properties, as more protein can migrate to the air-water interface, thereby increasing FC and FS (Yue et al., 2022). DES-CG provides better FC than A9 and moderate FS and ES, suggesting these solvents moderately alter protein structure, enhancing air trapping and emulsion formation while maintaining stability. Similarly, the DES-extracted oat protein was suggested to have higher foaming capacity and stability (Yue et al., 2021). DES methods provide preferable structural modification for functional properties without severely compromising stability.

## 4 Conclusion

The study demonstrates the efficacy of different extraction methods, namely alkaline extraction (A9) and deep eutectic solvent methods (DES-CG and DES-CL), in extracting proteins from MS. Among the methods, A9 resulted in the highest protein content and extraction yield but a lower protein recovery rate, indicating a less efficient protein recovery. Conversely, DES-CG and DES-CL, although demonstrating lower extraction yields, achieved higher protein recovery rates, highlighting their selective and protein-preserving extraction capabilities. FTIR analysis confirmed the formation of DESs and their efficiency in protein extraction, with DES-CG and DES-CL showing strong protein bands indicative of substantial protein extraction. Morphological analysis via SEM further supported the superior refinement and uniformity of DES-extracted proteins compared to alkaline, with DES methods showing specific variations in surface porosity due to solvent interactions. The solubility analysis showed that the DES methods preserved protein solubility, effectively disrupted the lignocellulosic matrix and improved protein release. The emulsion properties showed that MS powder retained the highest emulsifying activity and stability, while both DES methods slightly outperformed A9, indicating that DES extraction is less damaging to protein structure. The DES-CG method produces a greater foam capacity than alkaline extraction, indicating that DES solvents may better preserve or enhance structures that promote foaming. Furthermore, the foam stability of both DES-CL and A9 is superior to that of DES-CG, reflecting a more favorable balance between foam capacity and stability. Overall, the results highlight the potential of DES methods as sustainable and efficient alternatives for extracting proteins from lignocellulosic biomass, balancing yield, solubility, and functionality. However, further research on functional performance, potential toxicity, and residual effects is needed before industrial application. Optimizing extraction processes is crucial for maximizing protein yield and functionality and enhancing the utilization of alternative protein sources in various applications.

## CRediT authorship contribution statement

**Gulsah Karabulut:** Writing – original draft, Visualization, Project administration, Methodology, Funding acquisition. **Deniz Günal Köroğlu:** Writing – review & editing, Visualization, Methodology. **Hao Feng:** Writing – review & editing. **Zekeriya Karabulut:** Methodology, Formal analysis.

## Declaration of competing interest

The authors declare that they have no known competing financial interests or personal relationships that could have appeared to influence the work reported in this paper.

## Data Availability

Data will be made available on request.
